# Autotaxin and Lysophosphatidic Acid Circulating Levels Correlate with Body Mass Index in Obese Subjects with MASLD

**DOI:** 10.3390/ijms27062548

**Published:** 2026-03-10

**Authors:** Rossella Tatoli, Leonilde Bonfrate, Caterina Bonfiglio, Pasqua Letizia Pesole, Dolores Stabile, Rossella Donghia, Giovanni De Pergola, Gianluigi Giannelli

**Affiliations:** 1 Unit of Data Science, National Institute of Gastroenterology IRCCS “Saverio de Bellis”, Castellana Grotte, 70013 Bari, Italy; leonilde.bonfrate@uniroma5.it (L.B.); catia.bonfiglio@irccsdebellis.it (C.B.); rossella.donghia@irccsdebellis.it (R.D.); 2 Department of Human Sciences and Promotion of the Quality of Life, San Raffaele Roma University, 00166 Rome, Italy; 3 Core Facility Biobank, National Institute of Gastroenterology IRCCS “Saverio de Bellis”, Castellana Grotte, 70013 Bari, Italy; letizia.pesole@irccsdebellis.it (P.L.P.); dolores.stabile@irccsdebellis.it (D.S.); 4 Center of Nutrition for the Research and the Care of Obesity and Metabolic Diseases, National Institute of Gastroenterology IRCCS “Saverio de Bellis”, Castellana Grotte, 70013 Bari, Italy; giovanni.depergola@irccsdebellis.it; 5 Scientific Direction, National Institute of Gastroenterology IRCCS “Saverio de Bellis”, Castellana Grotte, 70013 Bari, Italy; gianluigi.giannelli@irccsdebelli.it

**Keywords:** MASLD, autotaxin, lysophosphatidic acid

## Abstract

Scientific evidence supports the role of the autotaxin-lysophosphatidic acid (ATX-LPA) pathway in obesity and liver damage. The present study aim is to investigate variations in serum ATX and LPA levels across different BMI categories in a subcohort of subjects with MASLD. The study sample comprises 199 patients with liver steatosis from the most recent follow-up of the MICOL study, a prospective cohort study established in 1985, based on a random sample of the population of Castellana Grotte. In adjusted model, a positive association of BMI with ATX was observed when modeled as both a continuous (β = 0.018, *p* < 0.001, 0.012 to 0.024 95% C.I.) and categorical variable β = 0.170, *p* < 0.001, 0.111 to 0.228 95% C.I.). Conversely, a negative association was observed for LPA alone (β = −0.083, *p* = 0.020, −0.152 to −0.013 95% C.I.) and for the BMI and LPA interaction term (β = −0.109, *p* = 0.002, −0.176 to −0.042 95% C.I.). A positive association between ATX levels and BMI was found, whereas LPA levels tended to decrease with increasing BMI. Within the obese subgroup, ATX concentrations were notably higher in female compared to male participants. These findings suggest that elevated ATX in MASLD may reflect obesity-related metabolic and inflammatory alterations rather than adiposity alone, possibly involving altered LPA feedback and metabolism.

## 1. Introduction

Metabolic dysfunction-associated steatotic liver disease (MASLD) is currently considered the leading cause of chronic liver disease worldwide. It affects 30% of the adult population, with a rapidly increasing prevalence, in parallel with the rising rates of obesity and obesity-related diseases [1]. In Italy, 20–40% of the general population (approximately 1 in 2–5 individuals) is affected by hepatic steatosis; these figures increase markedly among obese and diabetic patients, reaching prevalence rates of up to 50–90% [2].

MASLD is characterized by the presence of liver steatosis in association with at least one metabolic risk factor (i.e., overweight and obesity, hypertension, type 2 diabetes, elevated cholesterol, fasting hyperglycemia) and in the absence of significant alcohol consumption [3].

Liver steatosis has long been recognized as the hepatic manifestation of metabolic syndrome.

The term MASLD was introduced in 2023 with Delphi’s consensus to replace the term “non-alcoholic fatty liver disease” (NAFLD) and emphasize the close relationship between fatty liver and metabolic syndrome [2,3,4]. The onset and progression of MASLD are influenced by both modifiable and non-modifiable factors and involve several molecular pathways [5,6].

Although age, sex, dietary habits, and genetic factors contribute to the development of liver steatosis, obesity, and insulin resistance (IR), they represent the strongest risk factors [7].

MASLD is closely related to obesity, with which it shares several pathogenic mechanisms [8]. Adipose tissue can contribute to varying degrees to the development and progression of MASLD [9]. For example, it has been observed that visceral adipose tissue is associated with greater insulin resistance at the hepatic level, higher lipid content in the liver, and increased release of pro-inflammatory and pro-fibrogenic molecules [10].

Adipose tissue functions as an endocrine organ, secreting bioactive molecules, known as adipokines, which play a key role in metabolic homeostasis, insulin sensitivity, and systemic inflammation [11]. Some adipokines are also involved in regulating inflammation and fibrogenesis in fatty liver disease. Studies on individuals with liver steatosis have shown that circulating adipokine levels reflect disease severity [12]. Great interest is being directed in particular towards the implication that two markers, autotaxin (ATX) and lysophosphatidic acid (LPA), have on liver disease. The ATX–LPA signaling axis is upregulated in metabolic conditions such as obesity and clinical studies have demonstrated elevated serum ATX levels in patients with chronic liver disease, correlated with the severity of steatosis, fibrosis, and systemic inflammation [13].

ATX is an adipokine with lysophospholipase D activity that catalyzes the hydrolysis of lysophosphatidylcholine (LPC) into lysophosphatidic acid (LPA) [14].

Circulating LPA levels are closely related to the ATX expression and/or enzymatic activity. LPA is both a structural glycerophospholipid component of cell plasma membranes [15] and a potent bioactive signaling molecule [16,17].

The best-known signaling functions of LPA are mediated through its extracellular binding to G protein-coupled receptors (LPA1–6), which display distinct tissue-specific expression patterns [13]. Recent studies have analyzed the role of the ATX-LPA pathway in obesity and IR [18].

In obese mouse models, a deficiency or pharmacological inhibition of ATX has been associated with the improvement in glucose tolerance [19]. Similarly, in obese humans, ATX mRNA expression in visceral adipose tissue is associated with reduced glucose tolerance [20]. Other studies have reported a strong association between ATX and LPA levels and markers of liver fibrosis [21]. Other studies have reported a strong association between ATX and LPA levels and markers of liver fibrogenesis, highlighting the well-established role of the ATX–LPA pathway in hepatic fibrogenesis. Although there is substantial evidence supporting the role of the ATX–LPA pathway in obesity and liver damage, the relationship between circulating levels of ATX and LPA across various phenotypes of metabolic dysfunction is not yet fully understood [22].

Notably, only a limited number of studies have evaluated ATX and LPA simultaneously, despite their roles in the same metabolic pathway [22,23].

The present study aims to investigate the association of serum ATX and LPA levels across different BMI categories within a subcohort of subjects with MASLD.

## 2. Results

The epidemiological and clinical characteristics of both the total cohort and the cohort stratified by BMI value are shown in Table 1. Our cohort is represented by a majority of male subjects (62.31%) with a mean age of 56.01 ± 6.42 years. The cohort was quite homogeneous in terms of sex, age, smoking habits, adherence to the Southern Mediterranean diet (rMED), marital status, job occupation, education, diabetes, and hypertension (*p* > 0.05). Conversely, clinical parameters, such as systolic blood pressure (SBP) and diastolic blood pressure (DBP), were significantly higher in obese subjects (Body Mass Index, BMI ≥ 30) compared to non-obese subjects (129.88 ± 14.84 vs. 122.26 ± 14.67, 82.93 ± 8.01 vs. 78.85 ± 7.89, *p* < 0.001). A similar result was observed for other variables, including waist (107.89 ± 10.68 vs. 89.43 ± 8.10, *p* < 0.001), glucose (102.56 ± 17.93 vs. 96.52 ± 13.08, *p* = 0.007), homeostasis model assessment (HOMA-IR) (4.75 ± 2.73 vs. 2.96, *p* < 0.001), aspartate amino transferase (AST), alanine amino transferase (ALT) (25.02 ± 12.77 vs. 22.02 ± 6.08, 30.33 ± 16.50 vs. 24.26 ± 10.52, *p* = 0.03 and *p* = 0.002, respectively), ferritin (164.19 ± 157.31 vs. 126.61 ± 102.02, *p* = 0.04), and autotaxin levels (ATX) (246.00 ± 66.18 vs. 197.86 ± 45.29, *p* < 0.001). In contrast, high-density lipoprotein cholesterol (HDL) levels were significantly lower in the same groups (45.06 ± 11.39 vs. 48.81 ± 13.23, *p* = 0.04).

The same analysis was also conducted after stratifying the cohort by sex, as detailed in Supplementary Table S3. Male participants were younger than female participants (54.98 ± 6.60 vs. 57.71 ± 5.74 years, *p* = 0.003) and were more frequently married or cohabiting (96.77% vs. 74.67%, *p* < 0.001). Additionally, men were significantly more often engaged in agricultural occupations (*p* < 0.001). From a clinical perspective, DBP was higher in men than in women (81.73 ± 8.12 vs. 78.53 ± 7.92, *p* = 0.007), likely reflecting the significantly greater body weight and waist circumference observed among men (86.08 ± 15.75 vs. 73.54 ± 13.19; 100.65 ± 12.37 vs. 91.06 ± 11.71, both *p* < 0.001). With respect to hepatic metabolism, ALT and GGT levels were significantly higher in men than in women (29.21 ± 14.71 vs. 22.72 ± 10.46, *p* < 0.001; 27.85 ± 34.99 vs. 18.25 ± 14.86, *p* = 0.025, respectively), as was ferritin concentration (180.83 ± 143.13 vs. 78.05 ± 60.01, *p* < 0.001). In contrast, men exhibited significantly lower levels of HDL (43.82 ± 10.99 vs. 52.96 ±13.11), platelet count (229.65 ± 47.62 vs. 257.79 ± 55.80), and LPA (192.42 ± 42.47 vs. 259.48 ± 60.68; all *p* < 0.001).

Table 2 presents the association between ATX, expressed as a natural logarithm, and BMI, modeled both as a continuous or categorical variable, as well as log-transformed LPA and their relative interactions. Each predictor was entered separately into the models, which were progressively adjusted for covariates (Models 1, 2, and 3). In the model, positive associations were found when BMI was included as a continuous variable (β = 0.021, *p* < 0.001, 0.015 to 0.028 95% C.I.) and as a categorical variable (β = 0.213, *p* < 0.001, 0.145 to 0.280 95% C.I.), as well as for the interaction between BMI and LPA (β = 0.003, *p* = 0.008, 0.001 to 0.006 95% C.I.). In model 2, adjusted for age and sex, these associations remained significant for BMI modeled as continuous (β = 0.019, *p* < 0.001, 0.014 to 0.025 95% C.I.) and categorical (β = 0.187, *p* < 0.001, 0.131 to 0.242 95% C.I.). Furthermore, a negative association was found for LPA (β = −0.075, *p* = 0.037, −0.146 to −0.005 95% C.I.), as well as for the interaction between BMI and LPA (β = −0.100, *p* = 0.004, −0.166 to −0.033 95% C.I.). These associations and trends were confirmed in Model 3, with BMI remaining positively associated with ATX when modeled as both a continuous (β = 0.018, *p* < 0.001, 0.012 to 0.024 95% C.I.) and categorical variable (β = 0.170, *p* < 0.001, 0.111 to 0.228 95% C.I.). Conversely, a negative association was observed for LPA alone (β = −0.083, *p* = 0.020, −0.152 to −0.013 95% C.I.) as well as for the interaction term between BMI and LPA (β = −0.109, *p* = 0.002, −0.176 to −0.042 95% C.I.).

Using the same covariate adjustment, the models were also stratified by sex (Supplementary Table S4). In Model 1, BMI modeled as both a continuous and categorical variable was positively associated with ATX in both females and males (*p* < 0.001). In addition, a negative association for LPA was observed only in females (β = −0.124, *p* = 0.029, −0.236 to −0.013). Conversely, a significant interaction between continuous BMI and LPA was detected in males (β = 0.003, *p* = 0.014, 0.001 to 0.006), whereas in females the interaction was significant only when BMI was modeled as a categorical variable (β = −0.151, *p* = 0.006, −0.259 to −0.044).

In the models adjusted for age (Model 2) and for additional covariates (Model 3), these associations were largely confirmed, with the exception of the interaction term of BMI/LPA, which did not retain statistical significance.

**Figure 1:**
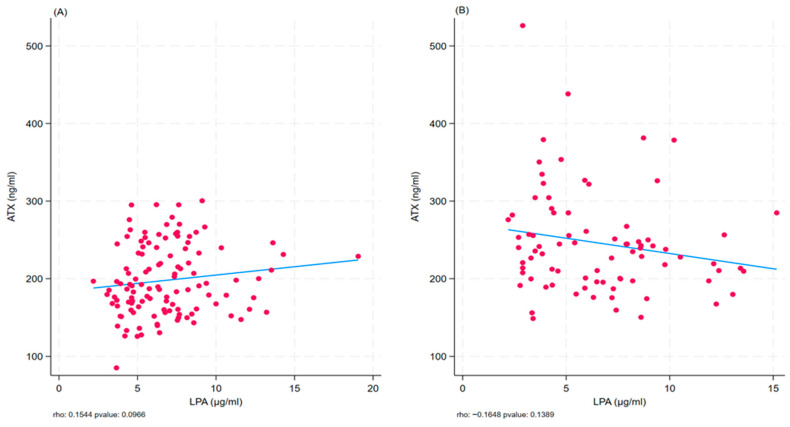
Scatter plot of ATX and LPA stratified by BMI in non-obese (**A**) and obese (**B**) subjects. Red dots represent the values of the two variables for a single observation. The blue line identifies the αSex-stratified scatter were also generated (Supplementary Figure S1A,B), showing a borderline association in the female subcohort (*p* = 0.06).

Scatter plots were generated to further investigate the strength and direction of the association between ATX or LPA and BMI across BMI subgroups (Figure 1A,B). Although these analyses did not reach statistical significance, a consistent trend was observed (*p* = 0.10 and *p* = 0.14, respectively).

## 3. Discussion

In this study, we evaluated the association between serum ATX and LPA levels across BMI categories in a cohort of subjects with MASLD in Southern Italy. Our findings show a positive association between ATX levels and BMI, while LPA levels tended to decrease as BMI increases. Specifically, obese subjects exhibited higher circulating levels of ATX but lower levels of LPA than non-obese subjects. In accordance with certain previous studies [24], we hypothesize that the divergence observed between ATX and LPA levels represents a compensatory feedback mechanism. In this mechanism, elevated LPA initially suppresses ATX expression, while lower LPA may stimulate ATX production via adipose tissue responses and systemic inflammatory signaling. This hypothesis is consistent with previous mechanistic data suggesting regulatory feedback between circulating LPA and ATX synthesis. From their results, Benesch et al. conclude that plasma LPA concentrations suppress the expression and secretion of ATX from adipose tissue. Conversely, decreasing plasma LPA concentrations increases ARX secretion by adipose tissue, resulting in higher plasma ATX levels, likely due to the regulatory role of inflammatory cytokines such as TNF-αor interleukin 1β [24].

Another interesting finding observed in our group of obese individuals is that ATX concentrations were higher in females relative to males. Consistent with our ATX findings, Reeves et al. reported a significant positive correlation between serum ATX, BMI, and waist circumference in older, non-diabetic, overweight, or obese subjects [25]. As in our study, ATX levels were higher in women than in men. This gender difference is explained by the sexually dimorphic nature of ATX, which is higher in females than in males [26,27].

In contrast to the results of our study, which found lower levels of LPA in the obese group, a study conducted in Poland on 100 healthy volunteers observed a positive association between LPA levels and BMI, with higher plasma LPA levels in obese and overweight subjects [28]. Michalcyk et al. explain this result by referring to the role played by LPA in the pathophysiology of obesity. The ATX–LPA signaling axis is engaged in inhibiting the expansion of adipose tissue [28]. In humans, obesity is associated with higher ATX expression in visceral adipose tissue [20]. The study by Dusaulcy et al. on transgenic mice carrying a null ATX allele specifically in adipose tissue (FATX-KO) showed enhanced nutritional fattening and about 40% reduced plasma LPA compared to wild-type mice [29]. This observation suggests that adipose tissue may influence plasma LPA concentration.

Studies conducted over the last 10 years have highlighted the important role played by the ATX-LPA axis in regulating energy and carbohydrate homeostasis and insulin activity, thereby contributing to the onset of alterations in glucose metabolism, insulin resistance, inflammation, mitochondrial dysfunction, tissue fibrosis, and hepatic steatosis [30]. Emerging evidence from mechanistic NAFLD models indicates that hepatocyte-derived ATX, rather than adipose tissue alone, may significantly contribute to disease progression via the suppression of PPARα and downstream metabolic regulators such as FGF21, thereby linking hepatic autotaxin production to steatosis and fibrosis pathways [31]. Vikrant P. Rachakonda et al. investigated autotaxin levels in women with liver steatosis and severe obesity. ATX levels were higher in subjects with NAFLD. They were positively associated with indicators of glucose homeostasis and liver function but not with markers of adiposity such as weight, BMI, and waist circumference, although ATX is produced and secreted by adipocytes [22]. This result may not be entirely consistent with ours, as we found ATX values to be positively associated with changes in BMI. However, the link with hepatic steatosis remains, as our study sample was MASLD.

The positive correlation between ATX, alkaline phosphatase, fasting blood glucose, fasting serum insulin, and HOMA-IR suggested that circulating ATX levels were more dependent on obesity-related disorders in glucose homeostasis and/or insulin action than on adiposity per se [22]. For this reason, from our point of view, it is interesting to study the ATX-LPA pathway within the broader context of MASLD.

Inflammation, alterations in glucose homeostasis, and insulin resistance are mechanisms that may be responsible for ATX overexpression in obesity and related metabolic diseases [30]. Among pro-inflammatory cytokines, interleukin 6 (IL-6) can induce insulin resistance and promote ATX expression in adipocytes via glycoprotein 130 signaling (gp130). Gp130 signals through the Janus kinase (JAK)-signal transducer and activator of transcription 3 (STAT3) axis to promote ATX expression in adipocytes [32,33,34]. Oral administration of the gp130 inhibitor in obese-insulin-resistant mice reduced ATX expression in the adipose tissue and the plasma ATX content [34]. Therefore, this study suggests that gp130 is required for robust ATX expression in adipocytes and for the upregulation of ATX in adipose tissue during obesity-insulin resistance.

Tumor necrosis factor α (TNFα), another pro-inflammatory cytokine, has also been shown to compromise insulin sensitivity, promoting increased expression and secretion of ATX in adipocytes [17]. Insulin appears to play a direct role in regulating ATX [35,36].

Secreted ATX activity was increased by insulin in a concentration-dependent manner, an effect that was PI3Kinase-dependent but mTORC1-independent [36]. Upregulation of ATX mRNA levels and ATX protein secretion by a 24 h and 48 h insulin treatment, respectively, was also observed in human adipose tissue explants [36].

Our study benefits from a relatively large human MASLD cohort and the simultaneous measurement of ATX and LPA, providing novel insights into their interrelationship across BMI categories. The study sample is also homogeneous and representative of both genders.

This study’s limitations include the cross-sectional design and reliance on ultrasound rather than MRI or biopsy to assess the severity of steatosis.

Future research should incorporate advanced imaging, LPA species profiling, and longitudinal designs to validate and extend our findings, particularly in diverse MASLD populations with variable degrees of inflammation, fibrosis, and cardiometabolic risk.

## 4. Materials and Methods

### 4.1. Study Population

The MICOL study (Italian Multicenter Cholelithiasis Study) is an ongoing prospective cohort study established in 1985, based on a random sample of the population of Castellana Grotte (Apulia, Italy) aged 30 years or older. Participants were followed up in 1992–1993, 2005–2006, and 2017–2020; the last follow-up was interrupted due to COVID-19 restrictions. The primary objective of the study was to estimate the prevalence of cholelithiasis and its associated risk factors, as well as to investigate other liver diseases [37]. Retrospective analysis also revealed a high prevalence of hepatitis C infection and NAFLD [38].

For the present study, we evaluated 199 patients with confirmed MASLD and obesity. from the most recent follow-up.

The Micol study was conducted in accordance with the ethical standards of the institutional research committee of the National Institute of Gastroenterology and Research Hospital “S. de Bellis” in Castellana Grotte, Italy (DDG-CE-589/2004, 18 November 2004).

### 4.2. Data Collection

In all study waves, trained staff collected data using structured questionnaires and standardized procedures. Socio-demographic, anthropometric, nutritional, health-related, and lifestyle information was recorded. Blood pressure was measured according to international guidelines [39].

Anthropometric measurements (i.e., weight, height, and waist circumference) were obtained following standard procedures. Weight and height were measured using SECA instruments (Model 700 and Model 206, SECA, Hamburg, Germany).

Height and body weight were recorded using standardized tools, and the BMI was calculated and classified according to the criteria established by the National Institutes of Health and WHO criteria: underweight (BMI < 18.5 kg/m^2^); normal weight (BMI ≥ 18.5–24.9 kg/m^2^); overweight (BMI ≥ 25.0–29.9 kg/m^2^); obese (BMI ≥ 30 kg/m^2^). Waist circumference was assessed at the midpoint between the lower margin of the last palpable rib and the superior border of the iliac crest, using a horizontal measuring plane. Values ≥ 94 cm in men and ≥80 cm in women were used as thresholds to define abdominal obesity and elevated cardiometabolic risk [40].

Hepatic steatosis was assessed and classified by liver ultrasound examination (LUS) in all study waves [41]. Ultrasound results are summarized in Supplementary Tables S1 and S2. Liver examinations were performed using an Esaote MyLab70 XVG device with a 5 MHz Convex probe (Genoa, Italy). Participants were advised to fast prior to the ultrasound examination to optimize image quality.

FibroScan represents a non-invasive, cost-effective, and validated method for evaluating hepatic steatosis and fibrosis in individuals at increased risk [42]. Although liver biopsy remains the gold standard for the diagnosis of steatosis, fibrosis, and hepatic inflammation, ultrasound-based elastography performed with FibroScan offers a painless and comprehensive evaluation of liver status and is currently recommended as a first-line diagnostic tool [43]. Hepatic fat content was quantified through vibration-controlled transient elastography (VCTE) combined with the Controlled Attenuation Parameter (CAP) at 3.5 MHz. A CAP value >275 dB/m was adopted as the rule-in threshold for defining steatosis [44]. Liver stiffness measurement (LSM) was used to estimate hepatic fibrosis, applying cut-off values of 8 kPa for significant fibrosis and 12 kPa for advanced fibrosis (stage 3). In our sample, no participant exhibited LSM values indicative of advanced fibrosis or cirrhosis (LSM ≥ 12 kPa).

### 4.3. Biochemical Analyses

Fasting blood samples were collected in the early morning hours. Blood cell count was measured by fluorescence flow cytometry using the automatic hematology analyzer Sysmex XT-1000 (Dasit, Cornaredo, Milano, Italy). Serum samples were analyzed for fasting serum glucose (FSG), fasting insulin, triglycerides, total cholesterol, LDL-C, HDL-C, AST, ALT, GGT, and α1-antitrypsin using the COBAS 8000 autoanalyzer (ROCHE Diagnostic SPA, Monza, Italy).

Glycated hemoglobin (HbA1c) was measured with the Capillarys 3 OCTA capillary electrophoresis system (Sebia Italia S.r.l., Bagno a Ripoli, Florence, Italy).

Insulin resistance was calculated using the Homeostasis Model Assessment of Insulin Resistance (HOMA-IR), according to established methods [45].

Serum autotaxin levels were quantified using an ELISA assay according to the manufacturer’s instructions (Human ENPP-2/Autotaxin Immunoassay ELISA Kit, R&D Systems, Bio-Techne, Minneapolis, MN, USA). The minimum detectable concentration of human ENPP-2 ranged between 0.055 and 0.157 ng/mL.

Serum lysophosphatidic acid (LPA) levels were determined using an ELISA kit (Human Lysophosphatidic Acid ELISA kit, Cusabio, Houston, TX, USA) following the manufacturer’s instructions. The assay sensitivity was <3.9 ng/mL.

### 4.4. Statistical Analysis

Subjects’ characteristics are presented as means and standard deviations (M ± SD) for continuous variables and as frequencies and percentages (%) for categorical variables.

Continuous variables were compared using the Wilcoxon rank-sum test (Mann–Whitney), whereas categorical variables were analyzed using the Chi-Square test.

The Spearman rank correlation coefficient was used to evaluate the strength and direction of the association between ATX or LPA and BMI in total cohort and subcohorts. Scatter plots with fitted regression lines were generated to visually represent these relationships.

To address the non-normal distribution of ATX and LPA, a natural logarithm transformation was applied prior to statistical analyses.

Univariate and multivariate linear regression models were estimated with ATX as the dependent variable, and BMI (modeled both as a continuous and a categorical variable), LPA, and their interaction as predictors. Multivariate models were additionally totally adjusted for relevant covariates (i.e., age, sex, smoke, education, WBC, White Blood Cells; AST, Aspartate Amino Transferase; ALT, Alanine Amino Transferase; HOMA-IR, and LPA, Lysophosphatidic Acid). Coefficients and corresponding 95% confidence intervals (CIs) were reported.

A retrospective power analysis was performed to confirm that the sample size was adequate, supporting the reliability of the results.

Two-tailed *p*-values < 0.05 were considered statistically significant. All analyses were conducted using Stata Statistical Software: Release 19 (StataCorp LLC, College Station, TX, USA, 2025).

## 5. Conclusions

In this study, circulating autotaxin levels were observed in all BMI categories in subjects with hepatic steatosis associated with metabolic dysfunction, while lysophosphatidic acid levels showed an opposite trend, leading to the hypothesis of a dissociation of the ATX-LPA axis in individuals with obesity.

The high level of ATX in MASLD evident from our results may reflect metabolic and inflammatory alterations related to obesity rather than adiposity alone, possibly involving impaired feedback regulation and altered LPA metabolism. Sex-specific differences further support a sex-dimorphic regulation of ATX already discussed in the literature.

Overall, our findings identify ATX as a potential biomarker of metabolic dysfunction in MASLD and highlight the relevance of the ATX-LPA pathway in the pathophysiology of hepatic steatosis. Further longitudinal and mechanistic studies are needed to clarify its causal role and therapeutic potential.

## Figures and Tables

**Table 1:** Epidemiological and clinical characteristics of subjects with steatosis, stratified by BMI categories (≥30 vs. <30).

Parameters *	Total Cohort (*n* = 199)	BMI (Kg/m^2^)		*p* ^^^
		<30 (*n* = 117)	≥30 (*n* = 82)	
Sex (%)				0.22 ^†^
Female	75 (37.69)	40 (34.19)	35 (42.68)	
Male	124 (62.31)	77 (65.81)	47 (57.32)	
Age (years)	56.01 ± 6.42	55.65 ± 6.38	56.52 ± 6.48	0.35
Smoker (%)				0.43 ^†^
Never/Former	170 (85.43)	98 (83.76)	72 (87.80)	
Current	29 (14.57)	19 (16.24)	10 (12.19)	
rMED	8.12 ± 2.18	8.24 ± 2.36	7.90 ± 1.79	0.33
Marital Status (%)				0.42 ^†^
Single	9 (4.52)	5 (4.27)	4 (4.88)	
Married or Cohabiting	176 (88.44)	106 (90.60)	70 (85.36)	
Separated or Divorced	7 (3.52)	4 (3.42)	3 (3.66)	
Widower	7 (3.52)	2 (1.71)	5 (6.10)	
Job occupation (%)				0.26 ^†^
Managers & Professionals	23 (11.56)	17 (14.53)	6 (7.32)	
Craft, Agricultural, and Sales Workers	103 (51.76)	64 (54.70)	39 (47.56)	
Elementary Occupations	30 (15.07)	16 (13.67)	14 (17.07)	
Housewife	17 (8.54)	9 (7.69)	8 (9.76)	
Pensioneers	18 (9.04)	7 (5.98)	11 (13.41)	
Jobless	8 (4.02)	4 (3.42)	4 (4.88)	
Education (%)				0.08 ^†^
Primary & Secondary School	122 (61.3)	67 (57.26)	55 (67.07)	
High School	56 (28.14)	33 (28.20)	23 (28.05)	
Graduated	21 (10.55)	17 (14.53)	4 (4.88)	
Diabetes (%)				0.10 ^†^
No	105 (93.75)	63 (96.92)	42 (89.36)	
Yes	7 (6.25)	2 (3.08)	5 (10.64)	
Hypertension (%)				0.19 ^†^
No	68 (59.13)	43 (64.18)	25 (52.08)	
Yes	47 (40.87)	24 (35.82)	23 (47.92)	
SBP (mmHg)	125.40 ± 15.18	122.26 ± 14.67	129.88 ± 14.84	<0.001
DBP (mmHg)	80.53 ± 8.17	78.85 ± 7.89	82.93 ± 8.01	<0.001
Waist (cm)	97.04 ± 12.96	89.43 ± 8.10	107.89 ± 10.68	<0.001
Glucose (mg/mL)	99.01 ± 15.51	96.52 ± 13.08	102.56 ± 17.93	0.007
HbA1c (mmol/mol)	38.10 ± 7.03	37.72 ± 6.61	38.63 ± 7.59	0.37
HOMA-IR	3.70 ± 3.39	2.96 ± 3.62	4.75 ± 2.73	<0.001
AST (U/L)	23.26 ± 9.52	22.02 ± 6.08	25.02 ± 12.77	0.03
ALT (U/L)	26.76 ± 13.61	24.26 ± 10.52	30.33 ± 16.50	0.002
GGT (U/L)	24.23 ± 29.41	22.52 ± 18.16	26.67 ± 40.39	0.33
Total Cholesterol (mg/dL)	196.54 ± 38.27	198.56 ± 35.61	193.66 ± 41.83	0.38
HDL (mg/dL)	47.27 ± 12.61	48.81 ± 13.23	45.06 ± 11.39	0.04
LDL (mg/dL)	124.83 ± 34.49	126.50 ± 31.69	122.43 ± 38.25	0.42
Triglycerides (mg/dL)	122.91 ± 74.33	116.36 ± 76.33	132.26 ± 70.79	0.14
Ferritin (ng/mL)	142.10 ± 128.71	126.61 ± 102.02	164.19 ± 157.31	0.04
WBC (10^3^/uL)	6.51 ± 2.37	6.39 ± 2.84	6.68 ± 1.46	0.40
Platelets (10^3^/uL)	240.26 ± 52.53	236.51 ± 50.25	245.60 ± 55.49	0.23
α1AT (mg/dL)	184.87 ± 42.58	185.75 ± 43.70	183.62 ± 41.15	0.73
ATX (ng/mL)	217.69 ± 59.65	197.86 ± 45.29	246.00 ± 66.18	<0.001
LPA (µg/mL)	6.72 ± 2.94	6.84 ± 2.78	6.56 ± 3.16	0.51

* As Mean and Standard Deviation (M ± SD) for continuous variables, and Frequency and Percentage (%) for categorical. ^^^ Wilcoxon rank-sum tests for continuous variables, and ^†^ Chi-Square test for categorical. Abbreviations: BMI, Body Mass Index; rMED, Relative Mediterranean Diet; SBP, Systolic Blood Pressure; DBP, Diastolic Blood Pressure; HbA1c, Glycosylated Haemoglobin; HOMA, Homeostasis Model Assessment; AST, Aspartate Amino Transferase; ALT, Alanine Amino Transferase; GGT, Gamma Glutamyl Transferase; HDL, High-Density Lipoprotein Cholesterol; LDL, Low Density Cholesterol; WBC, White Blood Cells; α1AT, Alpha-1 antitrypsin; ATX, Autotaxin; LPA, Lysophosphatidic Acid.

**Table 2:** Regression models of log(ATX) on BMI or LPA, and interactions.

Parameters	β	*p*	95% C.I.
Model 1			
BMI (continuous)	0.021	<0.001	0.015 to 0.028
BMI (categorical)			
<30 [Ref.]	--	--	--
≥30	0.213	<0.001	0.145 to 0.280
log(LPA)	−0.024	0.571	−0.109 to 0.060
log(LPA)#BMI (continuous)	0.003	0.008	0.001 to 0.006
log(LPA)#BMI (categorical)			
<30	−0.058	0.157	−0.139 to 0.022
≥30	0.042	0.317	−0.041 to 0.126
Model 2			
BMI (continuous)	0.019	<0.001	0.014 to 0.025
BMI (categorical)			
<30 [Ref.]	--	--	--
≥30	0.187	<0.001	0.131 to 0.242
log(LPA)	−0.075	0.037	−0.146 to −0.005
log(LPA)#BMI (continuous)	0.002	0.099	−0.0003 to 0.004
log(LPA)#BMI (categorical)			
<30 [Ref.]	−0.100	0.004	−0.166 to −0.033
≥30	−0.015	0.664	−0.085 to 0.054
Model 3			
BMI (continuous)	0.018	<0.001	0.012 to 0.024
BMI (categorical)			
<30 [Ref.]	--	--	--
≥30	0.170	<0.001	0.111 to 0.228
log(LPA)	−0.083	0.020	−0.152 to −0.013
log(LPA)#BMI (continuous)	0.001	0.426	−0.001 to 0.003
log(LPA)#BMI (categorical)			
<30 [Ref.]	−0.109	0.002	−0.176 to −0.042
≥30	−0.032	0.358	−0.102 to 0.037

Models 1: Univariate, i.e., insert single in the models; Models 2: adjusted for age, and sex; Models 3: adjusted for age, sex, smoke, education, WBC, AST/ALT ratio, HOMA-IR, and LPA. Abbreviations: β, Coefficient; 95% C.I., Confidence Interval at 95%; BMI, Body Mass Index; WBC, White Blood Cells; AST, Aspartate Amino Transferase; ALT, Alanine Amino Transferase; LPA, Lysophosphatidic Acid.

## Data Availability

The original data presented in this study are openly available in FigShare at https://doi.org/10.6084/m9.figshare.31264630.

## Supplementary Materials

The following supporting information can be downloaded at: https://www.mdpi.com/article/10.3390/ijms27062548/s1.
