# Supplementary material for: Autotaxin and Lysophosphatidic Acid Circulating Levels Correlate with Body Mass Index in Obese Subjects with MASLD
Source: Int J Mol Sci. 2026 Mar 10;27(6):2548. doi: 10.3390/ijms27062548 (PMC13026522; doi:10.3390/ijms27062548)
Supplement: Supplementary file 1 [file ijms-27-02548-s001.zip › Paper ATX-LPA-BMI Supplementary Filedocx.pdf]

Supplementary Material

Supplementary Table S1. Qualitative ultrasound criteria for liver morphology evaluation.

**Dimension**	Normal	Increased	Reduced
**Margins**	Regular	Irregulars	--
**Ecostructure**	Homogeneous/Normoechogenic	Uneven	Hyperechogenic

Supplementary Table S2. Ultrasound scoring system for the assessment of hepatic steatosis severity.

**Contrast between liver and renal parenchyma**	**Ultrasound beam penetration**	**Vascular blurring (especially veins)**
Homogeneous echogenicity with no evident liver–kidney contrast (0)	Hepatic parenchyma clearly visible from the surface to the diaphragm (0)	Vascular structures clearly visible (0)
Mild discrepancy in hepatic–renal echogenicity (1)	Attenuation of the ultrasound beam with partial loss of visualization of deep liver structures or diaphragm (1)	Loss of visualization of vascular structures (1)
Wide discrepancy between hepatic and renal (2)	Marked attenuation of the ultrasound beam with loss of visualization of deep liver structures and diaphragm (2)	Vascular structures poorly visible or not visible (2)
***Steatosis Score**: Absent (0); Mild (1-2); Moderate (3-5); Severe (6)*		

Supplementary Table S3. Epidemiological and clinical characteristics of subjects with steatosis, stratified by sex.

Parameters *	Sex		p ^
	Female *(n=75)*	Male *(n=124)*	
BMI (Kg/m²)	29.88±5.12	29.29±5.11	0.43
BMI (%)			0.22 †
<30	40 (53.33)	77 (62.10)	
≥30	35 (46.67)	47 (37.90)	
Age (years)	57.71±5.74	54.98±6.60	0.003
Smoker (%)			0.70 †
Never/Former	65 (86.67)	105 (84.68)	
Current	10 (13.33)	19 (15.32)	
rMED	8.26±2.43	8.05±2.03	0.53
Marital Status (%)			<0.001 †
Single	6 (8.00)	3 (2.42)	
Married or Cohabiting	56 (74.67)	120 (96.77)	
Separated or Divorced	7 (9.33)	0 (0.00)	
Widower	6 (8.00)	1 (0.81)	
Job occupation (%)			<0.001 †
Managers & Professionals	4 (5.33)	19 (15.32)	
Craft, Agricultural, and Sales Workers	35 (46.67)	68 (54.84)	
Elementary Occupations	9 (12.00)	21 (16.93)	
Housewife	17 (22.67)	0 (0.00)	
Pensioneers	6 (8.00)	12 (9.68)	
Jobless	4 (5.33)	4 (3.22)	
Education (%)			0.19 †
Primary & Secondary School	52 (69.33)	70 (56.45)	
High School	17 (22.67)	39 (31.45)	
Graduated	6 (8.00)	15 (12.10)	
Diabetes (%)			0.49 †
No	46 (92.00)	59 (95.16)	
Yes	4 (8.00)	3 (4.84)	
Hypertension (%)			0.28 †
No	33 (64.70)	35 (54.69)	
Yes	18 (35.29)	29 (45.31)	

(continue)

Parameters *	Gender		p ^
	Female *(n=75)*	Male *(n=124)*	
SBP (mmHg)	123.87±15.13	126.33±15.20	0.27
DBP (mmHg)	78.53±7.92	81.73±8.12	0.007
Weight (kg)	73.54±13.19	86.08±15.75	<0.001
Waist (cm)	91.06±11.71	100.65±12.37	<0.001
Glucose (mg/mL)	97.84±17.35	99.72±14.31	0.41
HbA1c (mmol/mol)	39.07±6.95	37.51±7.03	0.13
HOMA	3.28±1.98	3.96±4.00	0.17
AST (U/L)	21.97±5.82	24.03±11.13	0.14
ALT (U/L)	22.72±10.46	29.21±14.71	<0.001
GGT (U/L)	18.25±14.86	27.85±34.99	0.025
Total Cholesterol (mg/dL)	200.29±37.91	194.27±38.47	0.28
HDL (mg/dL)	52.96±13.11	43.82±10.99	<0.001
LDL (mg/dL)	124.65±31.61	124.94±36.22	0.95
Triglycerides (mg/dL)	114.75±62.21	127.85±80.63	0.23
Ferritin (ng/mL)	78.05±60.01	180.83±143.13	<0.001
WBC (10^3/uL)	6.24±2.84	6.67±2.03	0.22
Platelets (10^3/uL)	257.79±55.80	229.65±47.62	<0.001
α1AT (mg/dL)	187.68±40.97	183.18±43.60	0.47
ATX (ng/ml)	7.14±3.04	6.47±2.85	0.11
LPA (μg/ml)	259.48±60.68	192.42±42.47	<0.001

* As Mean and Standard Deviation (M±SD) for continuous variables, and Frequency and Percentage (%) for categorical.

^ Wilcoxon rank-sum tests for continuous variables, and † Chi-Square test for categorical.

Abbreviations: BMI, Body Mass Index; rMED, Relative Mediterranean Diet; SBP, Systolic Blood Pressure; DBP, Diastolic Blood Pressure; HbA1c, Glycosylated Haemoglobin; HOMA, Homeostasis Model Assessment; AST, Aspartate Amino Transferase; ALT, Alanine Amino Transferase; GGT, Gamma Glutamyl Transferase; HDL, High-Density Lipoprotein Cholesterol; LDL, Low Density Cholesterol; WBC, White Blood Cells; α1AT, Alpha-1 antitrypsin; ATX, Autotaxin; LPA, Lysophosphatidic Acid.

Supplementary Table S4. Regression models of log(ATX) on BMI as continuous or categorical, stratified by sex.

Parameters	Sex					
	Female			Male		
	β	p	95% C.I.	β	p	95% C.I.
Model 1						
BMI *(continuous)*	0.016	<0.001	0.007 to 0.025	0.022	<0.001	0.015 to 0.028
BMI *(categorical)*						
<30 *[Ref.]*	--	--	--	--	--	--
≥30	0.166	<0.001	0.077 to 0.256	0.203	<0.001	0.131 to 0.275
log(LPA)	-0.124	0.029	-0.236 to -0.013	-0.030	0.513	-0.121 to 0.061
log(LPA)#BMI *(continuous)*	-0.0001	0.934	-0.003 to 0.003	0.003	0.014	0.001 to 0.006
log(LPA)#BMI *(categorical)*						
<30	-0.151	0.006	-0.259 to -0.044	-0.057	0.188	-0.141 to 0.028
≥30	-0.081	0.144	-0.191 to 0.028	0.042	0.352	-0.047 to 0.131
Model 2						
BMI *(continuous)*	0.016	<0.001	0.007 to 0.025	0.021	<0.001	0.014 to 0.028
BMI *(categorical)*						
<30 *[Ref.]*	--	--	--	--	--	--
≥30	0.165	<0.001	0.075 to 0.255	0.196	<0.001	0.124 to 0.267
log(LPA)	-0.123	0.033	-0.235 to -0.010	-0.05	0.252	-0.144 to 0.038
log(LPA)#BMI *(continuous)*	-0.001	0.964	-0.003 to 0.003	0.002	0.048	0.001 to 0.005
log(LPA)#BMI *(categorical)*						

<30	-0.150	0.007	-0.258 to -0.04	-0.07	0.094	-0.157 to 0.012
≥30	-0.08	0.152	-0.191 to 0.030	0.02	0.655	-0.070 to 0.111

(continue)

Parameters	Sex					
	Female			Male		
	β	p-value	95% C.I.	β	p-value	95% C.I.
Model 3						
BMI *(continuous)*	0.013	0.012	0.003 to 0.023	0.021	<0.001	0.013 to 0.028
BMI *(categorical)*						
<30 *[Ref.]*	--	--	--	--	--	--
≥30	0.122	0.015	0.025 to 0.220	0.186	<0.001	0.108 to 0.264
log(LPA)	-0.123	0.027	-0.231 to -0.015	-0.063	0.178	-0.155 to 0.029
log(LPA)#BMI *(continuous)*	-0.001	0.477	-0.004 to 0.002	0.002	0.199	-0.001 to 0.005
log(LPA)#BMI *(categorical)*						
<30	-0.147	0.009	-0.256 to -0.039	-0.086	0.054	-0.174 to 0.002
≥30	-0.097	0.080	-0.206 to 0.012	0.002	0.967	-0.091 to 0.095

Models 1: Univariate, i.e. insert single in the models; Models 2: adjusted for age, and sex; Models 3: adjusted for age, sex, smoke, education, WBC, AST/ALT ratio, HOMA, and LPA. Abbreviations: β, Coefficient; 95% C.I., Confidence Interval at 95%; BMI, Body Mass Index; WBC, White Blood Cells; AST, Aspartate Amino Transferase; ALT, Alanine Amino Transferase; LPA, Lysophosphatidic Acid.

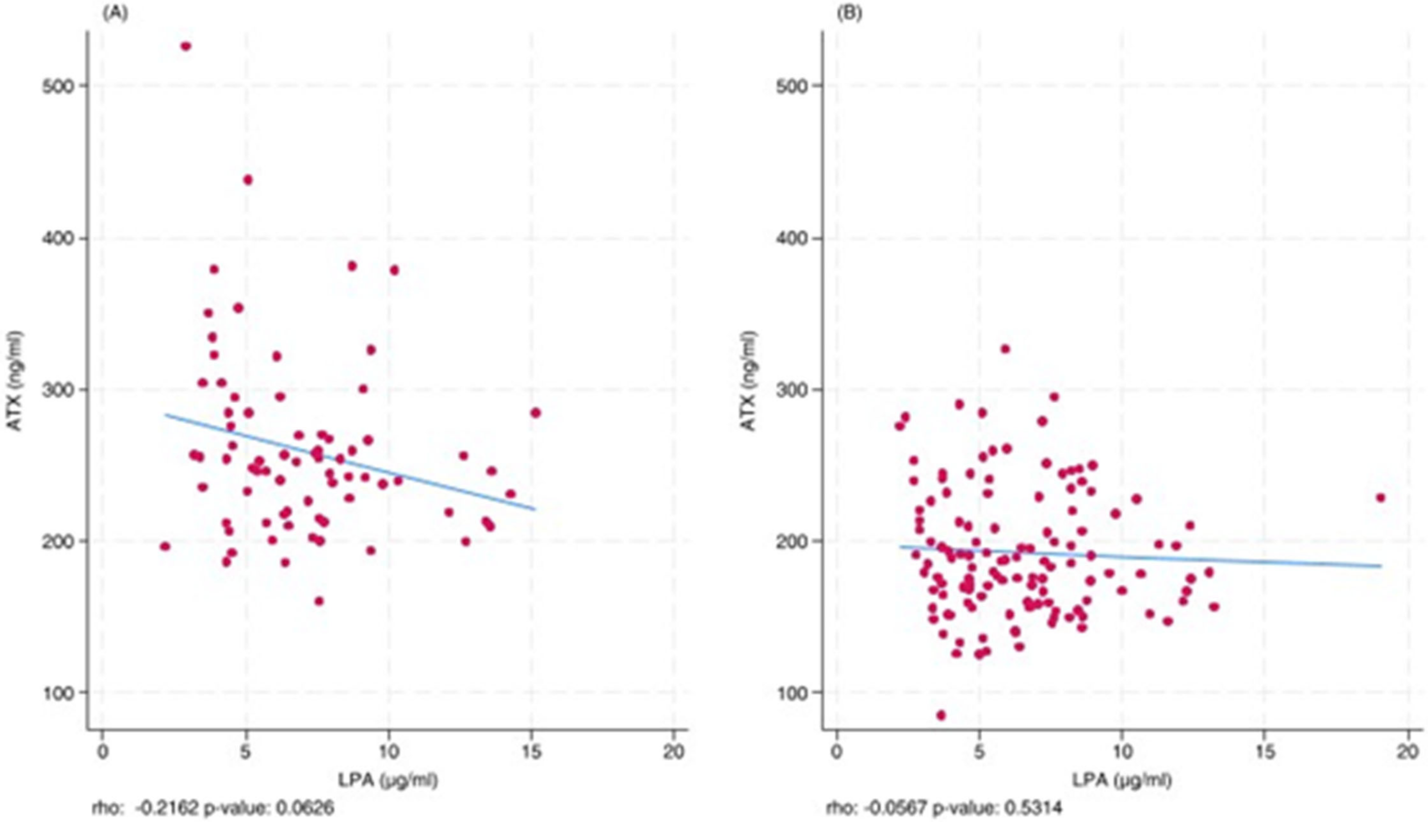

Supplementary Figure S1. Scatter plot of ATX and LPA stratified by sex, female (A) and male (B) subjects. Red dots represent the values of the two variables for a single observation. The blue line identifies the trend of the dots, the type and strength of the correlation between the two variables.
